# Involvement of the Catecholamine Pathway in Glioblastoma Development

**DOI:** 10.3390/cells10030549

**Published:** 2021-03-04

**Authors:** Zoltán Kraboth, Bela Kajtár, Bence Gálik, Attila Gyenesei, Attila Miseta, Bernadette Kalman

**Affiliations:** 1 Institute of Laboratory Medicine, School of Medicine, University of Pécs, 7624 Pécs, Hungary; zoltankraboth@gmail.com (Z.K.); miseta.attila@pte.hu (A.M.); 2 Szentagothai Research Center, University of Pécs, 7624 Pécs, Hungary; galik.bence@pte.hu (B.G.); gyenesei.attila@pte.hu (A.G.); 3 Institute of Pathology, School of Medicine, University of Pécs, 7624 Pécs, Hungary; kajtar.bela@pte.hu; 4 Department of Clinical Molecular Biology, Medical University of Bialystok, 15-267 Białystok, Poland

**Keywords:** DNA CpG methylation, gene expression, catecholamine pathway, sequential glioblastoma

## Abstract

Glioblastoma (GBM) is the most aggressive tumor of the central nervous system (CNS). The standard of care improves the overall survival of patients only by a few months. Explorations of new therapeutic targets related to molecular properties of the tumor are under way. Even though neurotransmitters and their receptors normally function as mediators of interneuronal communication, growing data suggest that these molecules are also involved in modulating the development and growth of GBM by acting on neuronal and glioblastoma stem cells. In our previous DNA CpG methylation studies, gene ontology analyses revealed the involvement of the monoamine pathway in sequential GBM. In this follow-up study, we quantitated the expression levels of four selected catecholamine pathway markers (alpha 1D adrenergic receptor—ADRA1D; adrenergic beta receptor kinase 1 or G protein-coupled receptor kinase 2—ADRBK1/GRK2; dopamine receptor D2—DRD2; and synaptic vesicle monoamine transporter—SLC18A2) by immunohistochemistry, and compared the histological scores with the methylation levels within the promoters + genes of these markers in 21 pairs of sequential GBM and in controls. Subsequently, we also determined the promoter and gene methylation levels of the same markers in an independent database cohort of sequential GBM pairs. These analyses revealed partial inverse correlations between the catecholamine protein expression and promoter + gene methylation levels, when the tumor and control samples were compared. However, we found no differences in the promoter + gene methylation levels of these markers in either our own or in the database primary–recurrent GBM pairs, despite the higher protein expression of all markers in the primary samples. This observation suggests that regulation of catecholamine expression is only partially related to CpG methylation within the promoter + gene regions, and additional mechanisms may also influence the expression of these markers in progressive GBM. These analyses underscore the involvement of certain catecholamine pathway markers in GBM development and suggest that these molecules mediating or modulating tumor growth merit further exploration.

## 1. Introduction

Glioblastoma (GBM) is the most aggressive and the most prevalent CNS tumor in adults, with a median survival of 15 months [1]. With the current standard of care (surgical removal with broad margins, radiation and chemotherapy), GBM invariably recurs and develops resistance to therapy. A better understanding of the molecular determinants of GBM is essential in the development of more efficient therapies.

Recently, we carried out a reduced representation bisulfite sequencing (RRBS) study to determine DNA CpG methylation genome-wide in 22 pairs of sequential GBM samples [2]. Gene ontology (GO) analysis revealed differential methylation in promoters and genes of several pathways, including hypomethylation in pathways of catecholamine secretion and transport in the primary compared to the recurrent tumor cohort. This observation suggested that catecholamines may predominantly contribute to early stages of GBM development. Based on the information from our RRBS methylome, here we selected four catecholamine markers differentially methylated at the cohort level (alpha 1D adrenergic receptor—ADRA1D; adrenergic beta receptor kinase 1 or G protein-coupled receptor kinase 2—ADRBK1/GRK2; dopamine receptor D2—DRD2; and synaptic vesicle monoamine transporter—SLC18A2; Supplementary Table S1a) [2], and analyzed further their involvement in GBM.

Catecholamines and their receptors are key elements of physiological interneuronal communication. However, a growing body of evidence suggests that these molecules also act on neuronal stem cells (NSC), progenitor cells (NPC) and glioblastoma stem cells (GSC), thereby promoting cell proliferation and differentiation in normal tissues and tumors such as GBM [3,4,5]. Nevertheless, data regarding the involvement of individual catecholamine markers are somewhat limited in GBM.

Of the four selected markers, ADRA1D is a member of the G protein-coupled receptor (GPCR) superfamily embedded in cell membranes in various organs. Its main ligand, norepinephrine (NE), is predominantly secreted by the locus coeruleus in the brain and by adrenal glands in the periphery. The alpha-1 adrenergic receptors activate the enzyme phospholipase C by G-protein dissociation, which hydrolyzes phosphatidylinositol 1,2-biphosphate, producing inositol trisphosphate (IP3) and diacylglycerol. These second messengers mediate intracellular Ca2+ release and activate protein kinase C, while also activating elements of other signaling pathways (e.g., voltage-dependent and independent calcium channels, arachidonic acid release, phospholipase A2, phospholipase D, and mitogen-activated protein kinase) [6]. The engagement of NE with ADRA1D initiates signals through these pathways in the sympathetic nervous system, modulates the contractions of the vascular smooth muscle cells, has trophic effects on endothelial cells [7,8,9], reduces insulin production [10], modulates the activity of lymphocytes [11], maintains tonic vessel tone and diverts blood flow to essential organs during fight-or-flight situations. These receptors are also important pharmacological targets in blood pressure management and regulation of urinary voiding [12]. In addition, ADRA1D engagement with NE also activates intracellular processes (e.g., by modulating the level of cyclic AMP) and regulates cell proliferation (e.g., by inhibiting mitogen-induced G1-S transition) [7,12,13,14]. In the periventricular germinal niches of the CNS, however, NE can determine the proliferative capacity of NPCs and negatively regulates periventricular neurogenesis [15]. NE and epinephrine (E) may promote cell migration and invasion in various cancers [16,17]. Activation of adrenoreceptors even by drugs can promote the development of certain cancers (e.g., gastrointestinal tract, liver) [7,18].

DRD2 also mediates its effects through G-proteins and induces pathways such as the mitogen-activated protein kinases/extracellular signal-regulated kinases (MAPK/ERK) or the phosphatidylinositol 3-kinase/protein kinase B (PI3K/Akt) signaling pathways involved in cell differentiation, growth, metabolism and apoptosis [19]. This receptor is localized in both the presynaptic and postsynaptic terminals [20,21]. Outside of the CNS, dopamine functions as a paracrine messenger, inhibits NE release and dilates blood vessels [22]. In the CNS, dopamine is predominantly secreted in the substantia nigra, ventral tegmental area and the arcuate nucleus of the hypothalamus [23]; it constitutes about 80% of the catecholamine content in brain. Under normal circumstances, dopamine participates in reward-motivated behavior, locomotion, memory, emotion and neuroendocrine control, and plays important roles in the brain circuitries involved in motor control [24,25]. Dysfunction in the dopaminergic neurotransmission caused by autoimmune, neurodegenerative or other pathologies, causes neurological and psychiatric disorders (e.g., Parkinson’s disease, Huntington’s disease, schizophrenia, dystonia, chorea) [19,26]. In addition, dopamine also regulates the development of γ-aminobutyric acidergic interneurons in the cerebral cortex and increases the production of new neurons in the hippocampus. These observations suggest that dopamine may promote proliferation and differentiation of the NSC and NPC populations [27,28]. Using a genome-wide shRNA screen, Li et al. [29] showed that the pathways of neurotransmitter receptor (i.e., DRD2) signaling are involved in GBM growth. DRD2 signaling may also induce functional changes and influence cell growth through an autocrine process in GBM [30,31].

ADRBK1/GRK2, like other G protein-coupled receptor kinases (GRK), is a modulator of signaling [32,33] through phosphorylation of GPCR, followed by the binding of arrestin proteins and uncoupling the receptors from G proteins, which lead to a clathrin-mediated receptor endocytosis and recycling [34,35]. GRKs, and GRK2 in particular, are expressed in different tissues (e.g., heart, liver, vessels) and influence numerous biological processes (e.g., insulin sensitivity, vasodilatation and vasoconstriction, lipogenesis and lipolysis, inflammation etc.) [36]. In addition to its classical role in promoting the desensitization and internalization of GPCRs, GRK2 may regulate non-GPCRs (e.g., through a direct association of GRK2 with the G beta-gamma complex [Gβγ], leading to desensitization of certain ion channels, or through GRK2-induced desensitization of the sphingosine-1-phosphate receptor [S1PR] that regulates lymphocyte migration into sites of infection). GRK2 also serves as a negative regulator of immune response via direct association with certain MAPK kinases (e.g., MEK) [37]. Further, GRK2 is capable of responding to non-receptor substrates to participate in cellular responses in a phosphorylation-independent manner (e.g., regulating microtubule assembly and agonist-induced GPCR internalization, thereby inducing actin cytoskeleton reorganization, inhibiting transforming growth factor-beta-mediated [TGF] cell growth arrest and apoptosis). [37,38,39]. By these functions, GRKs are involved in different signaling pathways contributing to angiogenesis, proliferation, migration and invasion of malignant tumors [40,41]. Changes in GRK expression or activity may promote oncogenic GPCR function [42]. Compared to low grade gliomas, GBM exhibits decreased GRK3 expression, resulting in a negative regulation of cell growth, and increased GRK5 expression, resulting in more aggressive tumor properties, particularly at recurrence [42,43]. GRKs, however, not only influence the biology of gliomas, but also have roles in other cancers [44].

The fourth marker involved in this study is SLC18A2 (also called the synaptic vesicle monoamine transporter [VMAT2]), a member of the solute carriers (SLCs). This is the largest family of transmembrane transporters; nevertheless, little is known about its exact function. What has been thus far established, is the involvement of these integral membrane proteins in the exchange of various nutrients, ions, metabolites and drugs across biological membranes [45]. In normal circumstances, SLC18A2/VMAT2 packages its cytosolic cargos (dopamine, norepinephrine, serotonin and histamine) into synaptic vesicles, releases them into the synaptic cleft and mediates their uptake. Differential expression distributions of SLC18A2/VMAT2 have been noted in various cell types (e.g., neurons vs. neuroendocrine cells). SLC18A2/VMAT2 has a neuroprotective effect to dopaminergic neurons in toxicity models by activating sequestration and removal of oxidized, neurotoxic dopamine molecules from the cytoplasm. Thus, SLC18A2 is essential for dopamine signaling by enabling exocytosis, but also maintaining cellular health [46]. Based on the example of dopamine signaling, changes in the presynaptic expression of these transporter proteins (e.g., due to their inhibition by drugs) can influence the release and reuptake of monoamines and their postsynaptic signaling [47,48].

Altogether, these data and our previous observations [2] confirm the involvement of catecholamine pathway molecules in GBM. Since our previous epigenomic analysis did not specifically focus on individual catecholamine markers in individual tumor samples and had no expression assessments, in the present study, we aimed to analyze further the involvement of the four selected markers (ADRA1D, ADRBK1/GRK2, DRD2 and SLC18A2) in GBM, by zooming into their promoter and gene CpG methylation data in comparison with their protein expression levels in sections dissected from the same blocks of sequential GBM specimens.

## 2. Materials and Methods

### 2.1. Subjects of the Study

The study was approved by the Regional Clinical Research Committee (Number: 7517 PTE 2018 and 2019) and was compliant with the Declaration of Helsinki. All patients passed away prior to the initiation of the study and the samples were left over from routine histological evaluations.

GBM and control samples were obtained between 1999 and 2017 at the Department of Pathology, School of Medicine, University of Pecs. The formalin-fixed, paraffin-embedded (FFPE) GBM blocks were surgically removed sequential specimens, while the histological controls (HC) included postmortem FFPE normal brain specimens (obtaining surgically removed normal control brain specimens are not feasible; other neurological control brain tissue FFPE specimens are also not available at our center). The histopathological diagnosis of GBM was established according to the recent World Health Organization guidelines [49] and samples were subjected to several rounds of quality selection [2]. These GBM specimens were also included in our previous DNA CpG methylation study [2]. However, of the 22 tumor pairs in the epigenomic analyses, only 21 pairs could be used here, because one pair of blocks had insufficient amount of tissue for the execution of immunohistochemistry (IHC).

Thus, the GBM cohort included 21 pairs of primary (GBM1) and recurrent GBM (GBM2). All tumors were isocitrate dehydrogenase-1 R132H (IDH1 R132H) mutation-negative de novo GBMs, from 14 male and 7 female patients (Supplementary Table S1b). The GBM1 samples were surgically obtained after the diagnosis, while the GBM2 tumor samples were removed at recurrence, after rounds of chemo- and irradiation therapy. All but one patient received temozolomide-based chemo- and radiation therapy after the first surgery. In the IHC study, six post-mortem FFPE samples from patients who died of non-neurological reasons were used (HC). Due to the high DNA fragmentation rates of these post-mortem samples (Supplementary Table S1c), we could not use them as controls in the DNA CpG methylation analyses. Therefore, in the epigenomic analyses methylation data of five brain specimens obtained during epilepsy surgery were included as the methylation controls (MC), by downloading the RRBS sequences from the EBI European genome–phenome archive (accession number: EGAS00001002538) [50].

### 2.2. DNA Isolation, Library Preparation, CpG Methylation Profiling and Bioinformatic Analyses

The DNA isolation and methylation profiling procedures were previously described in detail [2]. In brief, five 3–5 µm-thick cuts per FFPE block were used for DNA extraction with the QIAamp DNA FFPE Tissue Kit (Qiagen GbmH, Hilden, Germany). DNA quantitation was carried out by using the Qubit™ 1X dsDNA HS Assay Kit (Invitrogen, Carlsbad, CA, USA) on a Qubit 3 Fluorimeter (Invitrogen, Carlsbad, USA). Bisulfite converted libraries were prepared from DNA by using the Premium Reduced Representation Bisulfite Sequencing (RRBS) kit 24x (Premium RRBS Kit 24x, Diagenode SA, Seraing, Belgium). The amplified libraries were sequenced using the NextSeq 500/550 High Output Kit v2.5 (75cycles) (Illumina, San Diego, CA, USA) on a NextSeq 550 machine (Illumina, San Diego, CA, USA). After quality checking and filtering, the bisulfite-treated reads were aligned to the hg19 reference genome. After methylation calls by Bismark [51], RnBeads were used to identify the differentially methylated sites, regions and pathways in the control and GBM cohorts. Biological interpretation of data was assisted by the BioMethyl R package.

To assess in individual tumor and control samples the DNA CpG methylation levels within the promoters and genes of the markers selected based on the previous RnBeads GO analysis results [2], an in-house R script was applied. As the CpG methylation data in the promoter regions only (appr. 2kb) were insufficient for statistical analyses of differential methylation in samples (due to the quality of DNA from the FFPE blocks), we decided to examine the level of methylation in the promoter and gene region together (see the method in the Statistics section).

### 2.3. Immunhistochemistry (IHC)

From each of the 21 pairs of FFPE GBM blocks, 3–5 µm-thick sections were made. First, we determined the ROI in the tumor center based on hematoxylin–eosin staining, aiming to include (1) as much of the malignant tumor region as possible, defined by high degrees of cellularity, polymorphic nuclei and mitotic rates; and (2) little or no necrosis and vascularity. In a pilot study, we optimized the parameters for the retrieval of antigens and dilutions of the primary antibodies for the four catecholamine markers (Supplementary Table S1d). Secondary antibodies and the substrate diaminobenzidin (DAB) were used for labeling the primary antibody binding, all included in the Novolink Polymer Detection System RE7150-CE kit (Leica Biosystems, Newcastle, UK). For visualization of cell-nuclei, hematoxylin counter-staining was performed.

**Figure 1:**
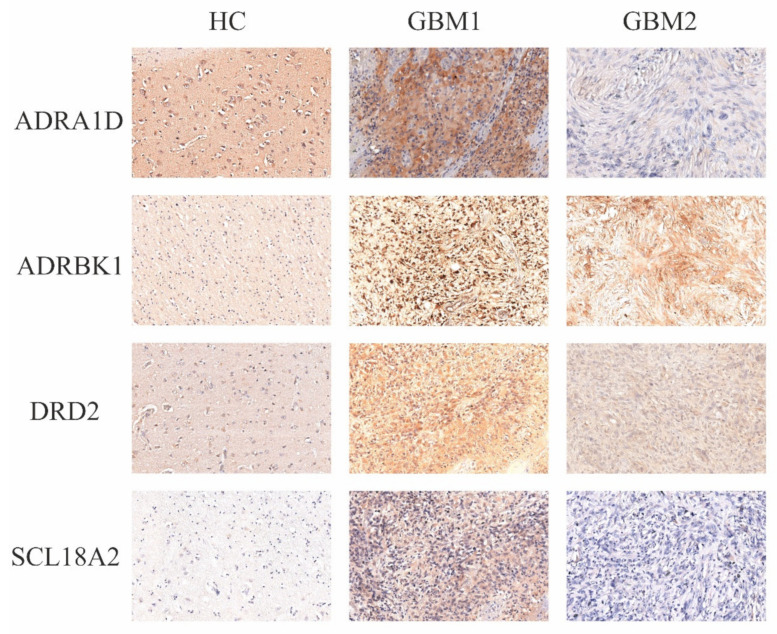
Representative staining for each marker. This figure shows representative images of the IHC preparations with the four selected markers, namely, ADRA1D, ADRBK1, DRD2 and SLC18A2, from the controls and a GBM sample (sample numbers 315 vs. 16534; 7442 vs. 16534; 7244 vs. 16534; 7166 vs. 16534, respectively. The GBM sample was a recurrent GBM).

The quantitative evaluation of IHC preparations was carried out manually, complemented by automated reading. Manual evaluations were made by three independent readers (the first, second and last authors), using a 1:200 magnification objective of a Nikon Optiphot-2 microscope. Within the ROI, we defined the intensity of staining on a scale of 0, +, ++ and +++ (in a numerical scale of 0, 1, 2 and 3), and the percentage of stained neoplastic cells. The complex score (CS) was derived by multiplying the numerical values of the staining intensities by the percentage of positive cells. Figure 1 presents representative images of the IHC staining for the four catecholamine markers. 

### 2.4. Statistics and Bioinformatics

For the intergroup comparisons of IHC CSs and CpG methylation data of the controls and GBM1 and GBM2, we applied the Mann–Whitney U test, while in cases of GBM pair comparisons, we used the Wilcoxon signed rank test. These statistical tests were ran using the SPSS v.26.0 package (SAGE, IBM^®^ SPSS^®^ Statistics v26.0). Bioinformatic analyses of DNA CpG methylation in sequential GBM has been detailed in our previous publication by Kraboth et al. [2], where the identification of the differentially methylated pathways was revealed at the cohort level. In short, FastQC was used in the quality control step, followed by filtering the sequences to remove low quality bases and adapters by TrimGalore applying RRBS specific parameters. Reads were aligned to the hg19 (GRCh37) reference genome and methylation calls were performed by Bismark. RnBeads was run to identify the differentially methylated pathways. In the present study, the quantitative methylation data of the four selected catecholamine markers in individual samples were extracted from the methylome data [2] using an in-house-generated R script. In brief, the script uses the individual data extracted from the Bismark methylation results and the human GRCh37 chromosome as inputs. Custom genomic ranges can be specified by the *chr*, *start* and *end* parameters. There is an option to define a prefix name in the output files and plots according to the genomic region of interest (gROI). There also is an option for paired samples, where the methylation patterns will be plotted as sample pairs. During the analysis, the first CpG data based on the defined gROI were extracted for all samples from the input files. Next, all possible CpG sites were identified within the gROI using the reference genome. Finally, the methylation levels in each sample are expressed in percentages, which were calculated from the captured number of methylated sites divided by all possible CpG sites detected within the gROI and multiplied by 100. Regions that contain 0 for all samples were removed from the results in order to simplify the plots. At the end, the script visualizes the methylation patterns for each sample or sample pair in PNG format, and separate tables contain the corresponding raw methylation data and methylation pattern data that were used for the visualization.

## 3. Results

### 3.1. Protein Expression Levels of the Four Catecholamine Markers in Paired GBM Samples and Controls

**Figure 2:**
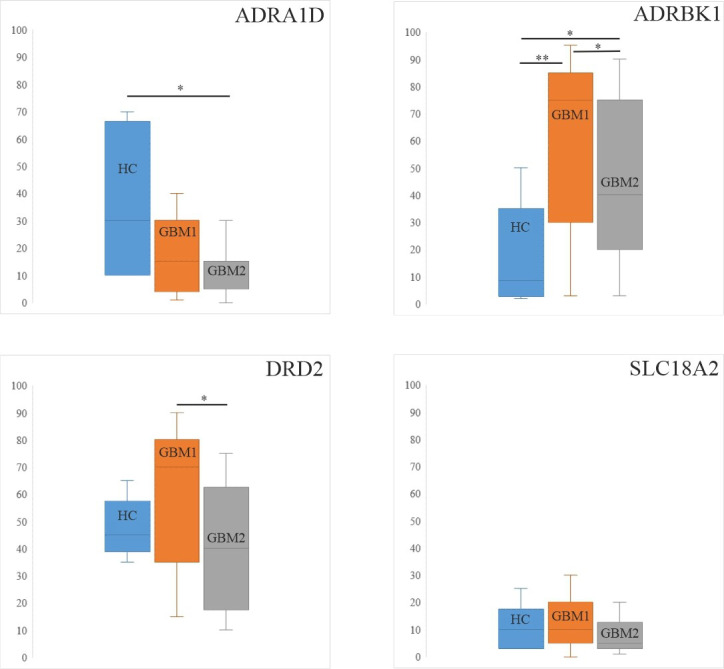
IHC complex score (CS) values of the markers in HC, GBM1 and GBM2. This figure shows the median and interquartile range values of the IHC complex scores (CSs) of selected markers in the healthy control (HC), GBM1 and GBM2 groups. The Wilcoxon singed rank test (GBM1 vs. GBM2) and Mann–Whitney-U test (HC vs. GBM1 or GBM2) *p*-values are indicated (* *p* < 0.05, ** *p* < 0.01).

We assessed the protein expression levels of the four catecholamine markers (ADRA1D, ADRBK1, DRD2 and SLC18A2) in 21 pairs of GBM1 and GBM2, and in 6 HC samples. Figure 1 shows representative IHC staining with the four markers in GBM and HC. In Figure 2, we present median and Q3–Q1 interquartile range (IQR) values of CS in HC, GBM1 and GBM2. In the control and GBM comparisons, ADRA1D was significantly lower in GBM2 (5 (15–5)) (*p* = 0.005) and tendentiously lower in GBM1 (15 (25–5)) compared to the HCs (30 (59–13)). In contrast, ADRBK1 was significantly higher in both GBM1 (75 (85–30)) (*p* = 0.004) and GBM2 (40 (75–20)) (*p* = 0.012) than in the HCs (9 (25–4)). There was a strong trend, which however, did not reach statistical significance, towards a higher expression of DRD2 in GBM1 (70 (80–40)) than in the HCs (45 (54–40)). 

In the comparisons of the sequential GBM pairs, the IHC evaluations showed significantly higher expressions of ADRBK1 (75 (85–30) vs. 40 (75–20)) (*p* = 0.011) and DRD2 (70 (80–40) vs. 40 (60–20)) (*p* = 0.026) in GBM1 compared to GBM2, respectively. ADRA1D (15 (25–5) vs. 5 (15–5)) and SLC18A2 (10 (20–5) vs. 5 (10–3)) HSs were tendentiously, but not significantly higher in GBM1 than in GBM2, respectively (Figure 2).

### 3.2. Promoter and Gene Methylation Profiles of the Four Catecholamine Markers in Individual GBM and Control Samples

**Figure 3:**
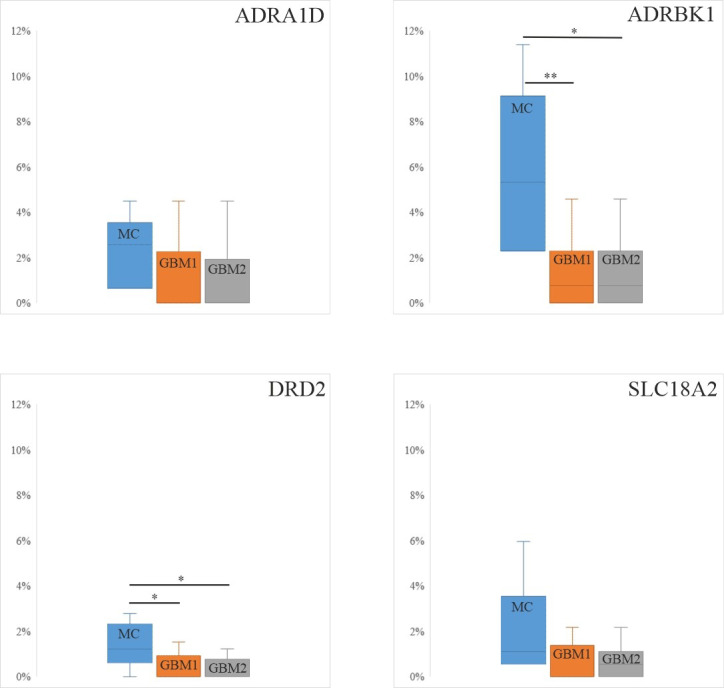
Promoter and gene methylation levels of the four markers in MC, GBM1 and GBM2. This figure presents the median and interquartile range values of the DNA CpG methylation levels in the promoters and genes of the selected markers in the methylation control (MC) (from Klughammer et al. 2018 [50]) and the GBM1 and GBM2 groups. The Wilcoxon singed rank test (GBM1 vs. GBM2) and Mann–Whitney-U test (HC vs. GBM1 or GBM2) *p*-values are indicated (* *p* < 0.05, ** *p* < 0.01).

First, we investigated the methylation levels within only the promoters of the selected four markers (ADRA1D, ADRBK1, DRD2 and SLC18A2) in the MC samples (from the cohort of Klughammer et al. [50]) and GBM1 and GBM2 samples, but could not get sufficient CpG coverage due to the DNA quality in the FFPE specimens. Therefore, we next assessed DNA CpG methylation in the combined promoter and gene regions for these markers, which did provide sufficient data for statistical analyses (Figure 3). No significant differential methylation levels were seen in cases of ADRA1D (2.56 (3.04–2.08)) and SLC18A2 (1.08 (2.30–0.95) in the MC and GBM1 (0 (1.44–0) and 0 (1.22–0)) or GBM2 (0 (1.44–0) and 0.54 (1.08–0)) comparisons. However, both ADRBK1 and DRD2 showed significantly lower methylation levels in GBM1 (ADRBK1: 0.76 (1.33–0); *p* = 0.006; DRD2: 0 (0.46–0); *p* = 0.041) and GBM2 (ADRBK1: 0.76 (1.71–0); *p* = 0.01; DRD2: 0 (0.15–0); *p* = 0.019) than in the MC (ADRBK1: 5.31 (7.97–4.56); DRD2: 1.22 (2.06–1.22)), in an inverse relationship with the protein expression in GBM1 and HC, respectively. No significant differences were found in the methylation levels of the four markers in GBM1 and GBM2, suggesting that their significantly (ADRABK1 and DRD2) or tendentiously (ADRA1D and SLC18A2) decreasing expressions in GBM2 compared to GBM1 may be related to mechanisms other than changes in CpG methylation (Figure 3). 

### 3.3. DNA CpG Methylation Levels in Promoter and Gene Regions of Catecholamine Markers in a Database GBM Cohort (Figure 4)

**Figure 4:**
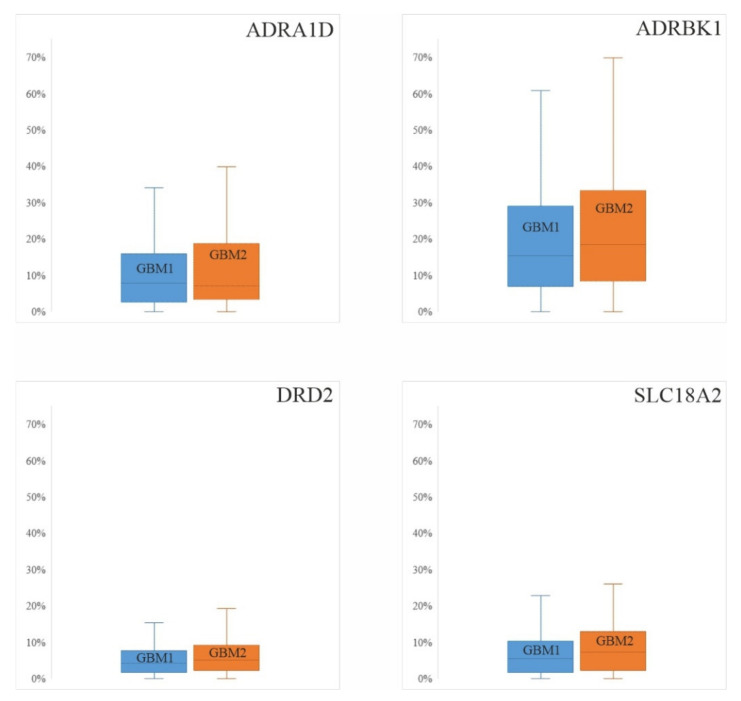
Promoter and gene methylation levels of the four markers in the database sequential GBM cohort. This figure depicts the median and interquartile range values of the DNA CpG methylation levels in the promoters and genes of the selected markers in a database of 112 sequential GBM pairs (Klughammer et al. 2018 [50]).

To validate our observations, we evaluated the same four catecholamine promoter and gene regions from the RRBS sequencing data of another sequential GBM cohort [50] (https://www.ebi.ac.uk/ena, accessed on 5 February 2021, Primary Accession: PRJEB38380, Secondary Accession: ERP121800). We could not compare DNA CpG methylation in the six controls and 122 GBM pairs because, by our method, the number of captured methylated CpG sites depended on the size of the cohort. To eliminate biases related to marked cohort size differences, we only compared the methylation data between the pairs of GBM. Similar to our own data, no differences were detected in the promoter and gene DNA CpG methylation levels when the primary and recurrent GBM pairs were compared (ADRA1D: 8.33 (16.66–3.84) and 8.01 (19.38–3.84); ADRBK1:17.46 (28.85–8.35) and 18.98 (33.40–9.87); DRD2: 4.59 (7.95–2.14) and 5.96 (9.17–2.75); SCL18A2: 5.96 (11.37–3.25) and 7.58 (13.00–3.25)) (Figure 4). This observation strengthens the notion that the differential protein expression levels detected by IHC in GBM1 and GBM2 are likely related to regulatory mechanisms beyond promoter and gene methylation. 

## 4. Discussion

This study is a follow-up to our recent DNA CpG methylation analyses [2] revealing lower promoter and gene methylation in the pathways of catecholamine secretion and transport in primary compared to recurrent GBM at the cohort level. As CpG hypo- or unmethylation allows the binding of transcription factors, and thereby the initiation of gene transcription and expression, our epigenomic observations suggested the involvement of this signaling pathway in GBM, but without identifying the involvements of individual markers and their expression levels. While several in vitro studies on various cancer and glioma cell lines have investigated the involvement of this pathway in tumorigenesis, we could not find related studies on human brain-derived GBM specimens where tumor cells had been under the influence of their microenvironment. These observations prompted us to determine here the protein expression and promoter + gene methylation levels of four selected catecholamine markers in individual samples of primary and recurrent GBM [2].

First, we performed quantitative IHC analyses of the four selected markers (Figure 2), which revealed that the levels of protein expression were either significantly (ADRBK1, DRD2) or tendentiously (ADRA1D, SCL18A2) higher in primary (GBM1) compared to recurrent tumors (GBM2). Comparing GBMs to HCs, ADRA1D decreased, while ADRBK1 increased in the tumors, and DRD2 and SLC18A2 did not show statistical differences in these comparisons. Second, we assessed the promoter + gene CpG methylation levels for the four selected markers in individual samples of the 21 GBM1 and GBM2 pairs, and also compared these figures to those of the MC group (five individuals) (Figure 3). We opted to determine the methylation levels of the corresponding promoter and gene regions together, instead of only the promoter regions of the markers, because of the compromised quality of DNA derived from the FFPE specimens. These analyses showed either significantly or tendentiously higher methylation levels of all four markers in the MC compared to the GBM1 and GBM2 samples. In cases of ADRBK1/GRK2 and DRD2, the differences were significant in both the MC vs. GBM1 and MC vs. GBM2 comparisons, in inverse correlations with the protein expressions of these markers in the HC and GBM1 comparisons. While we also expected to detect lower promoter + gene methylation levels when the protein expression levels were higher for the markers in the sequential GBM pairs, our data did not show such correspondence in the GBM1 and GBM2 comparisons (Figure 2 and Figure 3). In fact, there was no difference in methylation levels when the GBM1 and GBM2 samples were compared. Third, we wanted to validate our observation on the promoter + gene methylation levels in another cohort. Therefore, we assessed the methylation levels of the promoter + gene segments of the four markers in the RRBS sequence data of the sequential GBM pairs published by Klughammer et al. [50]. To avoid biases related to the marked cohort size differences, we only compared the 122 GBM pairs to each other, but not to the 5 MCs (in the RRBS sequence data, the cohort size greatly influences the detection of the number and methylation status of CpGs in a given region; when the sizes of compared cohorts greatly differ, biased outcome may be gained regarding CpG methylation differences) (Figure 4). These analyses showed no differences in the methylation levels of the four markers in the primary and recurrent GBM comparison in concordance with the outcome of our own sequential GBM1 and GBM2 comparisons.

The lack of lower promoter + gene methylation accompanying the significantly or tendentiously higher protein expression levels of the four markers in GBM1 compared to GBM2 may be explained by alternative mechanisms of gene expression regulation. Similarly, inverse correlations between protein expression and promoter + gene methylation measures were only observed for ADRBK1 and DRD2 in the GBM1 vs. control comparisons and for ADRBK1 in the GBM2 vs. control comparisons, further underscoring the involvement of other mechanisms besides promoter + gene CpG methylation in protein expression regulation. Methylation changes may affect not only a particular gene and promoter region, but also numerous molecules involved in gene expression regulation, binding sites of transcription factors, splice sites, or coding regions of microRNA and siRNA molecules [52]. Furthermore, newly acquired somatic structural chromosomal alterations, gene copy number variations, or changes in histone modification may also influence the protein expression patterns in sequential tumors [53]. This short list of various mechanisms influencing gene expression regulation may explain, at least in part, the lack of expected inverse relations between the detected protein expression and methylation data in our control, GBM1 and GBM2 cohorts. While this mechanistic complexity provides a strict control for gene expression regulation, it also makes it more difficult to achieve a targeted manipulation of critical molecules involved in pathological conditions such as tumorigenesis.

**Figure 5:**
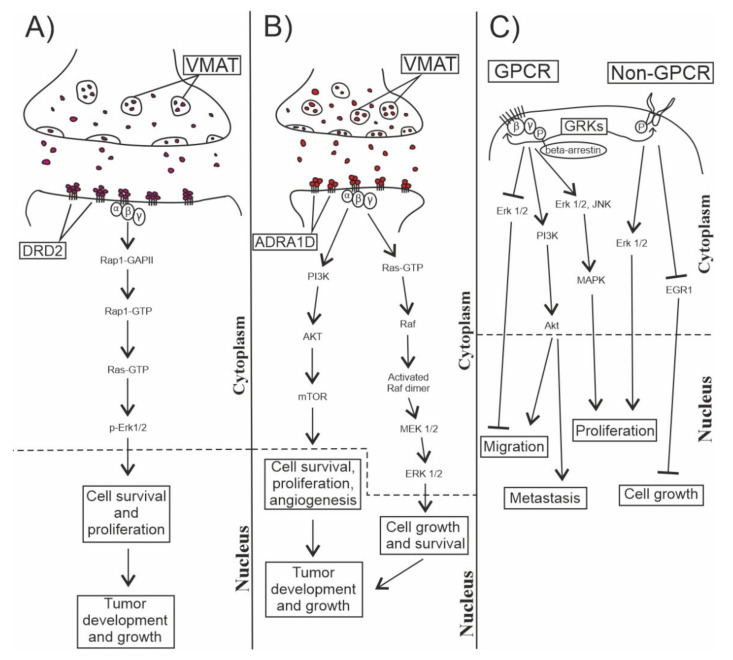
Involvements of the studied neurotransmitters in tumor development. This schematic figure illustrates as to how the signal transduction pathways of catecholamines and their associated receptors contribute to tumorigenesis. (**A**) Signaling pathway of dopamine via DRD2; (**B**) signaling pathway of norepinephrine or epinephrine via ADRA1D; and (**C**) interaction of GRKs with GPCRs and non-GPRCs to regulate cell biology and gliomagenesis. VMAT2 = vesicular monoamine transporter 2; GPCR = G protein-coupled receptor; GRK = G protein-coupled receptor kinase; DRD2 = dopamine receptor D2; ADRA1D = alpha-1D adrenoreceptor.

Nevertheless, our data support that catecholamines are differentially involved in progressive GBM samples compared to each other and to non-tumorous brain tissues. Figure 5 depicts signaling and potential effects of the studied markers in GBM. Catecholamines, including dopamine, epinephrine and norepinephrine, are important physiological neurotransmitters, mediating a variety of CNS functions (e.g., motor control, cognition, endocrine modulation) [54]. Neurons synthesize monoamines and deliver them to the synaptic cleft, from where many molecules diffuse out and activate autoreceptors on the surface of the emitting neurons or on the surrounding astrocytes and oligodendrocytes [55,56]. CNS cells transformed into tumor-initiating cells are also exposed to neurotransmitters. The higher DRD2 mRNA and protein levels in human neoplastic tissues than in normal brain controls [29,57], and the higher DRD2 levels in primary than in recurrent GBM (in the present study) support the hypothesis that dopamine has an influence on glioma formation and growth [29]. Single-cell RNA sequence data revealing an increased expression in the PI3K-Akt pathway genes that code for signaling molecules downstream to DRD2 provide indirect support to these observations in GBM, as the PI3K–Akt pathway is associated with increased cell differentiation and growth [58]. While these data suggest that dopamine may contribute to or modulate carcinogenesis by its pro-proliferative properties through DRD2, its effects are more complex. Exogenous administration of dopamine was reported to reduce angiogenesis and tumor growth in breast- and colon-cancer by inhibiting vascular endothelial growth factor (VEGF)-induced phosphorylation of VEGFR2 and its downstream pathway in mice [59]. Similarly, dopamine inhibited tumor growth, while levodopa, its precursor, increased the survival time of rats with C6 glioma [60]. Further, the repression of DRD2 signaling by an upstream regulator named repressor element-1 silencing transcription factor (REST) resulted in GSC-mediated tumorigenesis [61]. These observed opposing effects complicate the potential use of dopamine-targeting drugs in cancer. Our data on the GBM and control samples also did not reveal unequivocal direction of DRD2 expression changes, suggesting that further research is needed to better dissect the complex role of dopamine during gliomagenesis and progression in the setting of intra- and intertumor heterogeneity. GRKs (including ADRBK1) are a family of protein kinases that phosphorylate the intracellular domains of the GPCRs and thereby regulate the downstream G-protein signaling pathways. GRK3 is a known negative regulator of GBM, whereas GRK5 contributes to its more aggressive growth and drug resistance [42,43]. While specific data are unavailable about the role of GRK2 or ADRBK1 in GBM, several GRKs are known to influence cell proliferation and tumor growth in other types of cancers [44]. Although GRK2 knockdown slowed cell growth and proliferation, and enhanced apoptosis in UW228-2 medulloblastoma cell lines [62], its enhanced expression increased the sensitivity of adenosine A_2A_ receptor (ADORA2A) to desensitization in a mouse neuroblastoma x rat glioma construct [63]. As GPCR desensitization depends on the level of GRK expression, cells that express high levels of GRK2 are likely to trigger GRK-mediated pathways at low agonist concentrations [63]. These observations raise the possibility that ADRBK1/GRK2 and GPCRs (e.g., DRD2) may promote cell proliferation and tumor growth in GBM. Adrenergic neurotransmitters (NE, E) are known to promote cell migration and invasion in various types of cancer [16,17], and an abnormal activation of adrenoreceptors (e.g., by GRK feedback or drugs) may promote cancer development [7,18]. Further, ERK1/2 in U373 MG cells is also activated by several mitogenic GPCRs, including alpha-adrenergic receptors [64]. Although we found higher expression levels of ADRA1D in the HC group than in the GBM groups, we cannot rule out the possibility that changes in its expression may influence tumor formation. The vesicular monoamine transporters are expressed in dopaminergic, serotonergic and noradrenergic cells in the brain, and thus may be responsible for the storage and transport of all monoamines in the CNS [65,66]. While these transporters have roles in the regulation of neurotransmitter release and post-synaptic signaling, and their altered expression may confer risk for some neuropsychiatric disorders, their involvement in cancer remains equivocal at the present time [48]. Experiments with VMAT2 expressing mouse lines demonstrated that, following treatment with a dopaminergic toxicant (e.g., MPTP), the low level of VMAT2 caused catecholaminergic cell loss, while the high level of VMAT2 increased dopamine release and protected cells, observations that may predict what role this transporter plays in cancer [67]. While the effects of monoamine depletion in animal models and human studies on cognition (e.g., depression, decision making, etc.) are well defined [68,69], the data concerning the in vivo or in vitro effects of exogenously administered or depleted monoamines in GBM/glioma cell lines are less unequivocal. 

As the currently approved Stupp protocol [70] only prolongs patients’ life, but does not cure GBM, research on additional potential treatment targets are essential. In this line, a combined application of temozolomide and DRD2 antagonists was shown to have synergistic effects in inhibiting the proliferation of glioma cells [71]. Further, the epidermal growth factor receptor (EGFR), Ras-GTP, Erk1 and Erk2 pathway is positively modulated by dopamine signaling [29]. While targeting EGFR by monoclonal antibodies or tyrosine kinase inhibitors have been extensively studied, a combined blockade of the EGFR and DRD2 pathways remains to be tested. GRK2 has not been characterized or targeted in GBM, but is known to promote tumorigenesis and chemotherapy-induced apoptosis in breast [72] and medulloblastoma cells [62]. The modulation of the ADRA1D signaling in tumors also remains to be better explored. Some data show that inhibition of the ADRA1D, MAPK and PI3K-Akt pathway leads to increased death in myoblast cells [73], suggesting a contribution of ADRA1D to tumor cell survival; however, we could not find previous data about its role in GBM. Abnormal expression of SLC18A2 may also affect GBM cells and deserve further exploration as a treatment target. Recent observations support that neuronal activity fosters glioma cell malignancy by non-synaptic paracrine and autocrine mechanisms as well as by functional synapses between neurons and glioma cells [74,75]. These electrochemical connections can induce proliferation and progression of tumor cells involving neurotransmitters [75]. Altogether, our data and observations in the literature suggest that neurotransmitters, particularly catecholamines, are highjacked by GBM for its own benefit and growth, indicating a new direction for research on complementary treatment approaches (Figure 5). However, it is important to note that in vivo data concerning the roles of catecholamines in gliomas are scarce, and no previous human study had shown that this signaling pathway has a key importance in GBM development. Genome-wide transcriptome or single-cell RNA sequencing analyses, although identified numerous pathways and gene products in association with GBM [76,77,78], have not highlighted the importance of this pathway. Given the confounding effects of GBM inter- and intratumor heterogeneity, and the complexity of interactions between tumor cells and their microenvironment, in vivo or ex vivo capturing the impacts of the catecholamine pathway activation on glioma growth is not straightforward. Only integrated results of bulk tissue and single cell OMICS and imaging complemented by in vitro manipulation of ligands, receptors and signaling molecules may bring us closer to define if elements of this pathway may become worthy of therapeutic targeting in humans.

The strength of this study is that our cohort contained sequential GBM samples, and the DNA CpG methylation and protein expression levels were compared in the same specimens. While this study provides new and important information about the potential role of catecholamine pathway markers in GBM, it also has weaknesses. First, the relatively small size of the sequential GBM cohort posed limitations in the statistical analyses. However, due to the aggressiveness of the tumor, higher numbers of GBM pairs are seldom available even at major university centers. Second, the quality of the human clinical samples was greatly influenced by the FFPE procedure that yielded chemically compromised DNA. Fortunately, the availability of a newer technology based on RRBS allowed us to obtain useful information even from FFPE specimens. Third, obtaining surgically removed normal or non-tumorous brain tissues for the controls is not feasible. Using postmortem brain controls in IHC and epilepsy surgery controls in the CpG methylation studies was a compromise commonly taken in similar studies. Despite all the quantitative and qualitative obstacles associated with human brain tissue and tumor studies, we believe that our observations represent valid data on the roles of catecholamines in GBM development, and open new avenues for future research on their mechanistic involvement in gliomagenesis or tumor-microenvironment communication.

## 5. Conclusions

This study has demonstrated that all four of the selected catecholamine pathway markers have higher expression at the protein level, either tendentiously (ADRA1D, SLC18A2) or significantly (DRD2, ADRBK1/GRK2) in GBM1 compared to GBM2. Comparing the CpG methylation data of the promoter + gene segments of the selected markers, higher methylation levels were seen either significantly (ADRBK1, DRD2) or tendentiously (ADRA1D, SLC18A2) in the MC cohort compared to the GBMs, likely underlying, at least in part, the inverse protein expression detected in GBM1 vs. HC. However, the marker expression changes assessed by IHC were not accompanied by inverse changes in the promoter + gene methylation levels when GBM1 and GBM2 were compared, suggesting the involvement of regulatory mechanisms other than CpG methylation alone. The lack of methylation differences for the markers in our GBM1 and GBM2 cohorts was supported by similar data when the larger sequential cohort of Klughammer et al. [50] was tested. Altogether, these observations suggest that neurotransmitters, their receptors and mediators play important roles in gliomagenesis, likely driving onset, development and growth of GBM, and offer novel approaches to identify supplementary therapeutic targets. Despite the technical limitations, we believe that our results from human GBM are consistent with, but also extend, the existing data, and provide grounds for new research directions.

## Data Availability

Raw sequencing data were uploaded to the European Nucleotide Archive (https://www.ebi.ac.uk/ena, accessed on 5 February 2021, Primary Accession: PRJEB38380, Secondary Accession: ERP121800). Some of the data are also provided in the Electronic Supplementary Material.

## Supplementary Materials

The following are available online at https://www.mdpi.com/2073-4409/10/3/549/s1. Table S1: (a) GO analysis; (b) GBM Cohort; (c) Fragmentation data; (d) Antigens. https://szkk.pte.hu/hu/file/1609/download?token=fldUHgUY, accessed on 5 February 2021.

## Code Availability

Publicly available bioinformatics programs and tools were used as detailed in the Manuscript. The in-house script used in this study is available at https://github.com/galikbence/MethylPatternViz, accessed on 5 February 2021.

## References

[1] Alifieris C., Trafalis D.T. (2015). Glioblastoma multiforme: Pathogenesis and treatment. Pharmacol. Ther..

[2] Kraboth Z., Galik B., Tompa M., Kajtar B., Urban P., Gyenesei A., Kalman B. (2020). DNA CpG methylation in sequential glioblastoma specimens. J. Cancer Res. Clin. Oncol..

[3] Ligon K.L., Huillard E., Mehta S., Kesari S., Liu H., Alberta J.A., Anderson D.J. (2007). Olig2-regulated lineage-restricted pathway controls replication competence in neural stem cells and malignant glioma. Neuron.

[4] Zheng H., Ying H., Yan H., Kimmelman A.C., Hiller D.J., Chen A.J., Stommel J.M. (2008). p53 and Pten control neural and glioma stem/progenitor cell renewal and differentiation. Nature.

[5] Natsume A., Kinjo S., Yuki K., Kato T., Ohno M., Motomura K., Wakabayashi T. (2011). Glioma-initiating cells and molecular pathology: Implications for therapy. Brain Tumor Pathol..

[6] Lennarz W.J., Lane M.D. (2013). Encyclopedia of Biological Chemistry.

[7] Fitzgerald P.J. (2009). Is norepinephrine an etiological factor in some types of cancer?. Int. J. Cancer.

[8] Filippi S., Parenti A., Donnini S., Granger H.J., Fazzini A., Ledda F. (2001). α1D-adrenoceptors cause endothelium-dependent vasodilatation in the rat mesenteric vascular bed. J. Pharmacol. Exp. Ther..

[9] Vinci M.C., Bellik L., Filippi S., Ledda F., Parenti A. (2007). Trophic effects induced by α1D-adrenoceptors on endothelial cells are potentiated by hypoxia. Am. J. Physiol. Heart Circ. Physiol..

[10] Underland L.J., Mark E.R., Katikaneni R., Heptulla R. (2018). The impact of dopamine on insulin secretion in healthy controls. Indian J. Crit. Care Med. Peer Rev. Off. Publ. Indian Soc. Crit. Care Med..

[11] Sarkar C., Basu B., Chakroborty D., Dasgupta P.S., Basu S. (2010). The immunoregulatory role of dopamine: An update. Brain Behav. Immun..

[12] Michelotti G.A., Brinkley D.M., Morris D.P., Smith M.P., Louie R.J., Schwinn D.A. (2007). Epigenetic regulation of human α1d-adrenergic receptor gene expression: A role for DNA methylation in Spl-dependent regulation. FASEB J..

[13] Calzada B.C., De Artiñano A.A. (2001). Alpha-adrenoceptor subtypes. Pharmacol. Res..

[14] Meyer J.S., Quenzer L.F. (2005). Psychopharmacology: Drugs, the Brain, and Behavior.

[15] Weselek G., Keiner S., Fauser M., Wagenführ L., Müller J., Kaltschmidt B., Storch A. (2020). Norepinephrine is a negative regulator of the adult periventricular neural stem cell niche. Stem Cells.

[16] Entschladen F., Drell Iv T.L., Lang K., Joseph J., Zaenker K.S. (2004). Tumour-cell migration, invasion, and metastasis: Navigation by neurotransmitters. Lancet Oncol..

[17] Schuller H.M. (2007). Neurotransmitter receptor-mediated signaling pathways as modulators of carcinogenesis. Neuronal Activity in Tumor Tissue.

[18] Adissu H.A., Schuller H.M. (2004). Antagonistic growth regulation of cell lines derived from human lung adenocarcinomas of Clara cell and aveolar type II cell lineage: Implications for chemoprevention. Int. J. Oncol..

[19] Mishra A., Singh S., Shukla S. (2018). Physiological and functional basis of dopamine receptors and their role in neurogenesis: Possible implication for Parkinson’s disease. J. Exp. Neurosci..

[20] Burnett B.A., Womeldorff M.R., Jensen R. (2020). Meningioma: Signaling pathways and tumor growth. Handbook of Clinical Neurology.

[21] Quintana C., Beaulieu J.M. (2019). A fresh look at cortical dopamine D2 receptor expressing neurons. Pharmacol. Res..

[22] Armando I., Villar V.A.M., Jose P.A. (2011). Dopamine and renal function and blood pressure regulation. Compr. Physiol..

[23] Hegarty S.V., Sullivan A.M., O’Keeffe G.W. (2013). Midbrain dopaminergic neurons: A review of the molecular circuitry that regulates their development. Dev. Biol..

[24] Alcaro A., Huber R., Panksepp J. (2007). Behavioral functions of the mesolimbic dopaminergic system: An affective neuroethological perspective. Brain Res. Rev..

[25] Berridge K.C. (2007). The debate over dopamine’s role in reward: The case for incentive salience. Psychopharmacology.

[26] Ramanathan S., Al-Diwani A., Waters P., Irani S.R. (2019). The autoantibody-mediated encephalitides: From clinical observations to molecular pathogenesis. J. Neurol..

[27] Takamura N., Nakagawa S., Masuda T., Boku S., Kato A., Song N., Kusumi I. (2014). The effect of dopamine on adult hippocampal neurogenesis. Prog. Neuro-Psychopharmacol. Biol. Psychiatry.

[28] Ohira K. (2020). Dopamine as a growth differentiation factor in the mammalian brain. Neural Regen. Res..

[29] Li J., Zhu S., Kozono D., Ng K., Futalan D., Shen Y., Carter B.S. (2014). Genome-wide shRNA screen revealed integrated mitogenic signaling between dopamine receptor D2 (DRD2) and epidermal growth factor receptor (EGFR) in glioblastoma. Oncotarget.

[30] Caragher S.P., Shireman J.M., Huang M., Miska J., Atashi F., Baisiwala S., Lesniak M.S. (2019). Activation of dopamine receptor 2 prompts transcriptomic and metabolic plasticity in glioblastoma. J. Neurosci..

[31] Bartek J., Hodny Z. (2014). Dopamine signaling: Target in glioblastoma. Oncotarget.

[32] Métayé T., Gibelin H., Perdrisot R., Kraimps J.L. (2005). Pathophysiological roles of G-protein-coupled receptor kinases. Cell. Signal..

[33] Komolov K.E., Benovic J.L. (2018). G protein-coupled receptor kinases: Past, present and future. Cell. Signal..

[34] Ferguson S.S. (2001). Evolving concepts in G protein-coupled receptor endocytosis: The role in receptor desensitization and signaling. Pharmacol. Rev..

[35] Ribas C., Penela P., Murga C., Salcedo A., García-Hoz C., Jurado-Pueyo M., Mayor F. (2007). The G protein-coupled receptor kinase (GRK) interactome: Role of GRKs in GPCR regulation and signaling. Biochim. Biophys. Acta Biomembr..

[36] Murga C., Arcones A.C., Cruces-Sande M., Briones A.M., Salaices M., Mayor F. (2019). G protein-coupled receptor kinase 2 (GRK2) as a potential therapeutic target in cardiovascular and metabolic diseases. Front. Pharmacol..

[37] Evron T., Daigle T.L., Caron M.G. (2012). GRK2: Multiple roles beyond G protein-coupled receptor desensitization. Trends Pharmacol. Sci..

[38] Sun W.Y., Wu J.J., Peng W.T., Sun J.C., Wei W. (2018). The role of G protein-coupled receptor kinases in the pathology of malignant tumors. Acta Pharmacol. Sin..

[39] Penela P., Murga C., Ribas C., Lafarga V., Mayor F. (2010). The complex G protein-coupled receptor kinase 2 (GRK2) interactome unveils new physiopathological targets. Br. J. Pharmacol..

[40] Lymperopoulos A., Bathgate A. (2012). Pharmacogenomics of the heptahelical receptor regulators G-protein-coupled receptor kinases and arrestins: The known and the unknown. Pharmacogenomics.

[41] Nogués L., Palacios-García J., Reglero C., Rivas V., Neves M., Ribas C., Mayor F. (2018). G Protein-Coupled Receptor Kinases (GRKs) in Tumorigenesis and Cancer Progression: GPCR Regulators and Signaling Hubs. Seminars in Cancer Biology.

[42] Woerner B.M., Luo J., Brown K.R., Jackson E., Dahiya S.M., Mischel P., Rubin J.B. (2012). Suppression of G-protein–Coupled Receptor Kinase 3 Expression Is a Feature of Classical GBM That Is Required for Maximal Growth. Mol. Cancer Res..

[43] Kaur G., Kim J., Kaur R., Tan I., Bloch O., Sun M.Z., Parsa A.T. (2013). G-protein coupled receptor kinase (GRK)-5 regulates proliferation of glioblastoma-derived stem cells. J. Clin. Neurosci..

[44] Yu S., Sun L., Jiao Y., Lee L.T.O. (2018). The role of G protein-coupled receptor kinases in cancer. Int. J. Biol. Sci..

[45] Hu C., Tao L., Cao X., Chen L. (2020). The solute carrier transporters and the brain: Physiological and pharmacological implications. Asian J. Pharm. Sci..

[46] Mulvihill K.G. (2019). Presynaptic regulation of dopamine release: Role of the DAT and VMAT2 transporters. Neurochem. Int..

[47] Omote H., Miyaji T., Hiasa M., Juge N., Moriyama Y. (2016). Structure, function, and drug interactions of neurotransmitter transporters in the postgenomic era. Annu. Rev. Pharmacol. Toxicol..

[48] Lawal H.O., Krantz D.E. (2013). SLC18: Vesicular neurotransmitter transporters for monoamines and acetylcholine. Mol. Asp. Med..

[49] Louis D.N., Perry A., Reifenberger G., Von Deimling A., Figarella-Branger D., Cavenee W.K., Ellison D.W. (2016). The 2016 World Health Organization classification of tumors of the central nervous system: A summary. Acta Neuropathol..

[50] Klughammer J., Kiesel B., Roetzer T., Fortelny N., Nemc A., Nenning K.H., Nowosielski M. (2018). The DNA methylation landscape of glioblastoma disease progression shows extensive heterogeneity in time and space. Nat. Med..

[51] Krueger F., Andrews S.R. (2011). Bismark: A flexible aligner and methylation caller for Bisulfite-Seq applications. Bioinformatics.

[52] Li Z., Rana T.M. (2012). Molecular mechanisms of RNA-triggered gene silencing machineries. Acc. Chem. Res..

[53] Moarii M., Boeva V., Vert J.P., Reyal F. (2015). Changes in correlation between promoter methylation and gene expression in cancer. BMC Genom..

[54] Kobayashi K. (2001). Role of catecholamine signaling in brain and nervous system functions: New insights from mouse molecular genetic study. J. Investig. Dermatol. Symp. Proc..

[55] Xie Z., Westmoreland S.V., Bahn M.E., Chen G.L., Yang H., Vallender E.J., Miller G.M. (2007). Rhesus monkey trace amine-associated receptor 1 signaling: Enhancement by monoamine transporters and attenuation by the D2 autoreceptor in vitro. J. Pharmacol. Exp. Ther..

[56] Perea G., Navarrete M., Araque A. (2009). Tripartite synapses: Astrocytes process and control synaptic information. Trends Neurosci..

[57] Hottinger A.F., Stupp R., Homicsko K. (2014). Standards of care and novel approaches in the management of glioblastoma multiforme. Chin. J. Cancer.

[58] Pang B., Xu J., Hu J., Guo F., Wan L., Cheng M., Pang L. (2019). Single-cell RNA-seq reveals the invasive trajectory and molecular cascades underlying glioblastoma progression. Mol. Oncol..

[59] Sarkar C., Chakroborty D., Chowdhury U.R., Dasgupta P.S., Basu S. (2008). Dopamine increases the efficacy of anticancer drugs in breast and colon cancer preclinical models. Clin. Cancer Res..

[60] Qin T., Wang C., Chen X., Duan C., Zhang X., Zhang J., Yang J. (2015). Dopamine induces growth inhibition and vascular normalization through reprogramming M2-polarized macrophages in rat C6 glioma. Toxicol. Appl. Pharmacol..

[61] Marisetty A.L., Lu L., Veo B.L., Liu B., Coarfa C., Kamal M.M., Majumder S. (2019). REST-DRD2 mechanism impacts glioblastoma stem cell–mediated tumorigenesis. Neuro-oncology.

[62] Pathania A.S., Ren X., Mahdi M.Y., Shackleford G.M., Erdreich-Epstein A. (2019). GRK2 promotes growth of medulloblastoma cells and protects them from chemotherapy-induced apoptosis. Sci. Rep..

[63] Mundell S.J., Kelly E. (1998). The effect of inhibitors of receptor internalization on the desensitization and resensitization of three Gs-coupled receptor responses. Br. J. Pharmacol..

[64] Yamaguchi K., Kugimiya T., Miyazaki T. (2005). Substance P receptor in U373 MG human astrocytoma cells activates mitogen-activated protein kinases ERK1/2 through Src. Brain Tumor Pathol..

[65] Eiden L.E., Weihe E. (2011). VMAT2: A dynamic regulator of brain monoaminergic neuronal function interacting with drugs of abuse. Ann. N. Y. Acad. Sci..

[66] Fei H., Krantz D.E. (2009). Vesicular Neurotransmitter Transporters. Handbook of Neurochemistry and Molecular Neurobiology.

[67] Lohr K.M., Chen M., Hoffman C.A., McDaniel M.J., Stout K.A., Dunn A.R., Miller G.W. (2016). Vesicular monoamine transporter 2 (VMAT2) level regulates MPTP vulnerability and clearance of excess dopamine in mouse striatal terminals. Toxicol. Sci..

[68] Berman R.M., Sanacora G., Anand A., Roach L.M., Fasula M.K., Finkelstein C.O., Charney D.S. (2002). Monoamine depletion in unmedicated depressed subjects. Biol. Psychiatry.

[69] Schweimer J., Saft S., Hauber W. (2005). Involvement of catecholamine neurotransmission in the rat anterior cingulate in effort-related decision making. Behav. Neurosci..

[70] Stupp R., Mason W.P., Van Den Bent M.J., Weller M., Fisher B., Taphoorn M.J., Curschmann J. (2005). Radiotherapy plus concomitant and adjuvant temozolomide for glioblastoma. N. Engl. J. Med..

[71] Liu Z., Jiang X., Gao L., Liu X., Li J., Huang X., Zeng T. (2019). Synergistic suppression of glioblastoma cell growth by combined application of temozolomide and dopamine D2 receptor antagonists. World Neurosurg..

[72] Nogués L., Reglero C., Rivas V., Salcedo A., Lafarga V., Neves M., Zhou X.Z. (2016). G protein-coupled receptor kinase 2 (GRK2) promotes breast tumorigenesis through a HDAC6-Pin1 axis. EBioMedicine.

[73] Saini A., Al-Shanti N., Stewart C. (2010). C2 skeletal myoblast survival, death, proliferation and differentiation: Regulation by Adra1d. Cell. Physiol. Biochem..

[74] Venkataramani V., Tanev D.I., Strahle C., Studier-Fischer A., Fankhauser L., Kessler T., Horstmann H. (2019). Glutamatergic synaptic input to glioma cells drives brain tumour progression. Nature.

[75] Venkatesh H.S., Morishita W., Geraghty A.C., Silverbush D., Gillespie S.M., Arzt M., Woo P.J. (2019). Electrical and synaptic integration of glioma into neural circuits. Nature.

[76] Neftel C., Laffy J., Filbin M.G., Hara T., Shore M.E., Rahme G.J., Suvà M. (2019). An integrative model of cellular states, plasticity, and genetics for glioblastoma. Cell.

[77] Park J., Shim J.K., Yoon S.J., Kim S.H., Chang J.H., Kang S.G. (2019). Transcriptome profiling-based identification of prognostic subtypes and multi-omics signatures of glioblastoma. Sci. Rep..

[78] Yuan J., Levitin H.M., Frattini V., Bush E.C., Boyett D.M., Samanamud J., Sims P.A. (2018). Single-cell transcriptome analysis of lineage diversity in high-grade glioma. Genome Med..

